# Applying Neural Networks to Hyperspectral and Multispectral Field Data for Discrimination of Cruciferous Weeds in Winter Crops

**DOI:** 10.1100/2012/630390

**Published:** 2012-05-02

**Authors:** Ana-Isabel de Castro, Montserrat Jurado-Expósito, María-Teresa Gómez-Casero, Francisca López-Granados

**Affiliations:** Institute for Sustainable Agriculture (IAS), CSIC, P.O. Box 4084, 14080 Córdoba, Spain

## Abstract

In the context of detection of weeds in crops for site-specific weed control, on-ground spectral reflectance measurements are the first step to determine the potential of remote spectral data to classify weeds and crops. Field studies were conducted for four years at different locations in Spain. We aimed to distinguish cruciferous weeds in wheat and broad bean crops, using hyperspectral and multispectral readings in the visible and near-infrared spectrum. To identify differences in reflectance between cruciferous weeds, we applied three classification methods: stepwise discriminant (STEPDISC) analysis and two neural networks, specifically, multilayer perceptron (MLP) and radial basis function (RBF). Hyperspectral and multispectral signatures of cruciferous weeds, and wheat and broad bean crops can be classified using STEPDISC analysis, and MLP and RBF neural networks with different success, being the MLP model the most accurate with 100%, or higher than 98.1%, of classification performance for all the years. Classification accuracy from hyperspectral signatures was similar to that from multispectral and spectral indices, suggesting that little advantage would be obtained by using more expensive airborne hyperspectral imagery. Therefore, for next investigations, we recommend using multispectral remote imagery to explore whether they can potentially discriminate these weeds and crops.

## 1. Introduction

*Sinapis* spp and *Diplotaxis* spp are cruciferous weeds very abundant and competitive in temperate areas worldwide that reduce yield in winter cereal crops, such as wheat (*Triticum durum *L.) [1–3] and in legume crops such as lentil (*Lens culinaris *L.) [4, 5], broad bean (*Vicia faba *L.) [6], and pea (*Pisum sativum *L.) [7]. Although wheat sunflower is one of the main crop rotations in Spain, winter legume crops for human or animal consumption are also usually introduced into the crop rotations. The results of field surveys conducted recently on 30,000 ha near Córdoba and Seville in Andalusia, southern Spain, indicated that more than 65% of winter crops were infested with cruciferous weeds, including *Diplotaxis *spp. (generally *D. virgata* Cav. DC. and *D. muralis* L. DC) and *Sinapis* spp. (generally *S. arvensis *L. and* S. alba*) [8]. Winter crop weeds in cereals are often controlled by presowing herbicides (e.g., glyphosate) and applying preemergence herbicides to legumes (e.g., linuron in broad bean and pendimethalin in pea). However, these herbicides cannot adequately control cruciferous weeds and specific herbicides, such as triasulfuron, and can be applied to postemergence cereals at weed flowering stage, although postemergence herbicides for legume crops have not yet been developed, and thus tillage or hand weeding is frequently used to reduce cruciferous infestations. Moreover, most winter crops in Mediterranean conditions are produced with nontillage or minimal tillage techniques to reduce the impact of soil erosion. Consequently, weeds such as cruciferous have become more problematic because they cannot be reduced by repeated tillage or cultivation.

Despite the usual uniform management of fields, patchy distribution of cruciferous and other weed species, as well as the potential herbicide savings from treating only infested areas, has already been assessed using geostatistical approaches [9, 10]. However, herbicides are usually broadcast over entire fields, and there are evident economic and environmental risks from overapplication. To overcome this situation, patch spraying (in cereals) or hand weeding (in legumes) of cruciferous weeds has supported the feasibility of using site-specific weed management (SSWM) for control of these worldwide weeds. A key component of SSWM is precise and timely weed maps, and one of the crucial steps for weed mapping is weed monitoring, either by ground sampling or by remote detection and identification of weeds. The remote sensing of weed canopies can significantly improve reliability compared to ground visits only if the spectral and spatial resolutions of remote sensing equipment are sufficient for the detection of differences in spectral reflectance [11, 12]. Late-season weed detection maps can be used to design SSWM for the application of in-season postemergence herbicides in the case that adequate preemergence control was not achieved. Alternatively, because weed infestations are relatively stable from year to year, weed maps can be used for site-specific applications in subsequent years [13]. Both in-season and next-season maps are important for site-specific cruciferous weed control.

Spectral response of plant species at canopy or single leaf scale is unique and known as spectral signature. The basic principle of using ground-acquired spectral signatures of weeds and crops is that measured differences in reflectivity can be used to detect and distinguish different weed species either in real-time or for the creation of weed maps. Furthermore, these ground-acquired signatures can be used to build libraries for remote or proximal sensing. Differences in spectral reflectance can be highlighted by weeds' distinctive colours or phenological stages and by the use of vegetation indices [14–16]. Thus, there has been increased interest in the identification of weeds, soil background, and crops using their spectral signatures, along with powerful discrimination techniques, as a starting point for SSWM.

Hand-held hyperspectral sensors collect data in narrow and contiguous wavelengths, usually less than 10 nm wide, to allow detection of small or local variations in absorption features. By contrast, multispectral scanner systems collect data for several (3 to 7, usually 100 nm wide) broad bands, and, although multispectral data are typically easier to analyse, local variations in absorption might be undetectable within these broader bands. Hyperspectral and multispectral on-ground data have been successfully used to distinguish many plant groups, including 27 salt-marsh vegetation types in a coastal wetland [17]; corn caraway (*Ridolfia segetum *Moris.) in sunflower crop [14]; pitted morning glory (*Ipomea lacunosa *L.) [18]; grass weeds in wheat [16]; several weed species in common turfgrass [19]; volunteer potato and sugar beet [20]; five weeds and two crop species [21]. However, the use of hyperspectral recordings involves analysing hundreds of wavelengths, and it is necessary to use robust classification methods to select a subset of several wavelengths in order to reduce the large number of hyperspectral data without losing any important information. Neural networks are a powerful multivariate analysis tool that can detect significant spectral differences and classify the spectra of weeds and crops into specific groups. Neural networks have been successfully used for the spectral classification of grass weed species in winter wheat [15]; however, to the best of our knowledge, they have not yet been applied to cruciferous weeds.

Both hyperspectral and multispectral remote sensors have shown promise for weed mapping. The Compact Airborne Spectral Imager (CASI), an airborne hyperspectral sensor, is capable of acquiring data at up to 288 wavelengths in the visible and near-infrared spectral range (400–1000 nm) at 1.9 nm intervals, and, if proper altitudes are maintained, it can achieve spatial resolutions of 0.5 to 1 m, which are particularly useful for classifying vegetation classes. Additionally, the CASI spectral collection is user-programmable, which means that CASI imagery can be recorded at only a few programmed wavelengths, rather than the 288 available ones. The CASI sensor has successfully detected several grass and broadleaved weeds in soybean and corn fields using Fisher's linear or other discriminant analysis [22, 23] and using neural networks [24]. Multispectral and high-spatial-resolution satellite imagery is also capable of distinguishing weeds from crops. For example, QuickBird (average revisit 1–3.5 days; panchromatic image: 0.7-m pixel; multispectral image in the visible and near-infrared spectral range from 450 to 900 nm: 2.44-m pixel) has proven to have sufficient accuracy for mapping weeds, such as *Cirsium arvense* in sugar beet at the cotyledon stage [25].

The potential advantages and disadvantages for both remote platforms are as follows: (i) hyperspectral imagery is not yet available in many regions and is still expensive, whereas QuickBird imagery is cheaper and is available worldwide; (ii) QuickBird usually covers a larger surface area and could map weed patches in tens of infested fields, whereas hyperspectral airborne sensors usually cover a smaller area, although they have superior flight versatility. As part of an overall research programme to investigate the opportunities and limitations of remote sensed imagery in accurately mapping cruciferous weeds in winter crops, it is crucial to explore the potential of these two technologies to identify variations in weeds' hyperspectral and multispectral signatures across different years and locations. Such an approach should point out the significant variations in hyperspectral and multispectral signatures of the plant species studied, indicating a set of suitable wavelengths or wavebands for species discrimination.

Thus, our study had the following objectives: (i) to determine the hyperspectral and multispectral mean reflectance curves of cruciferous weeds and two winter crops (wheat and broad bean) in four years and different locations, (ii) to select the best hyperspectral wavelengths or multispectral wavebands to discriminate efficiently between vegetation types, (iii) to compare the accuracy performance for a spectrum classification into the specific group to which it belongs, and (iv) to establish the misclassification percentage. We aimed to identify suitable wavelengths for programming hyperspectral sensors such as CASI, as well as appropriate uses of multispectral QuickBird imagery for mapping cruciferous weeds in winter crops.

## 2. Materials and Methods

The study was conducted in Andalusia, southern Spain, in early spring from 2007 to 2010 at several locations near Córdoba and Seville. Fields were sown with wheat and broad bean crops, and all of them contained a natural mixture of cruciferous weed infestations (Table 1).

### 2.1. Spectral Readings

The spectral signatures of weed-free crop and cruciferous weed patches were taken using an ASD HandHeld FieldSpec spectroradiometer (Analytical Spectral Devices, Inc., 5335 Sterling Drive, Boulder, CO, USA) placed at a height of 60–80 cm above each plant species canopy. Winter wheat and broad bean crops showed the typical green colour of the vegetative growth stage, and cruciferous weeds displayed an intense yellow colour corresponding to the flowering growth stage, although cruciferous weeds from 2008 fields showed a lightly more advanced phenological stage and consequently they displayed a less bright yellow colour (adapted from [26]). 

 In each field, a total of 115 canopy spectral reflectance measurements were collected for each plant species along transects in order to characterise field variability. The spectral data were converted into reflectance, which is the ratio of energy reflected off the target to the energy that is incident on the target. Each spectral signature was calibrated using a barium sulphate standard reflectance panel as a reference (Spectralon, Labsphere, North Sutton, NH, USA) before and immediately after every ten measurements. Spectroradiometer readings were taken under sunny conditions between 12:00 h and 14:00 h local time [27] using a 25° field-of-view optic to measure an area of about 0.15 to 0.20 m^2^. Hyperspectral measurements were collected between 325 and 1075 nm with a bandwidth of 1.0 nm, although the reflectance spectra were noisy at the beginning and at the end of the range, and only the measurements between 400 and 900 nm were analysed. In addition, previous studies have shown that neighbouring wavelengths can frequently provide similar information. Thus, hyperspectral measurements were averaged to represent 100 5 nm wide measurements between 400 and 900 nm [14, 28], and these measurements were analysed statistically. Reflectance measurements at the canopy scale were also averaged to represent multispectral broad wavebands (blue, B: 450–520 nm; green, G: 521–600 nm; red, R: 630–690 nm; near-infrared, NIR: 760–900 nm), similar to those available on the commercial satellite QuickBird. The normalised difference vegetation index (NDVI = (NIR − R)/(NIR + R)) [29], the ratio vegetation index (RVI = NIR/R) [30], the R/B index [31] and other waveband ratios such as B/G, R/G; NIR/B; NIR/G were also calculated from the B, G, R, and NIR wavebands and analyzed. 

### 2.2. Discriminant Analysis

Hyperspectral, multispectral, and spectral vegetation indices data were subjected to discriminant analysis (DISCRIM) using SPSS software (SPSS 13.0, Inc., Chicago; Microsoft Corp., Redmond, WA). The basic problem in a discriminant analysis lies in assigning an unknown subject to one of two or more groups on the basis of a multivariate observation. The DISCRIM procedure permits the setting up of a predictive model of group membership based on characteristics observed in each case. The procedure originated a discriminant function, since the number generated corresponded to the number of groups minus one, based on linear combinations of the independent variables. The number of discriminant functions providing a statistically significant among-group variation essentially defined the dimensionality of the discriminant space. This test also measured the difference between groups [32]. To determine if the set of wavelengths (hyperspectral study) and wavebands and spectral vegetation indices (multispectral study) selected could be used to separate the three plant groups, stepwise discriminant function analyses (STEPDISC) were performed using SPSS. The STEPDISC procedure combined forward selection and backward elimination of the variables. Forward selection was employed for the inclusion of a variable, and backward elimination was used for the removal of variables no longer significant in the model [24]. For this study, a Wilks' lambda test was performed to determine the significance of each discriminant function. The Wilks' lambda values were indicative of the separability or discriminatory power of spectral wavelengths (i.e., the lower the Wilks' lambda value, the greater the spectral differentiation between groups [28]). At each step, the variable that minimised the overall Wilks' lambda was entered. In addition, the minimum partial F to enter a variable was 3.84, and the maximum partial F for removing a variable was 2.71 (more details in [33]). 

The STEPDISC model was calculated by considering cruciferous weeds, wheat, and broad beans as different classes. The functions were generated from a sample of cases for which their group membership was known (count data); these functions could subsequently be applied to new cases with measurements for the predictor variables but an unknown group membership. The suitability of the discriminant functions for a given classification was compared using a cross-validation method, which involves the calculation of misclassification matrices by determining the number of wrongly classified groups in any single class. The “one data out” approach for cross-validation was selected from the classification option of the STEPDISC analysis in order to assess the accuracy of the model. In the development of STEPDISC models, the data were divided into two parts. The first was used to develop and construct the model, while the second was used to validate its classification accuracy [32]. This method was applied to both reflectance values and spectral indices to construct a classification rule to discriminate between wheat, broad beans, and cruciferous weeds. 

### 2.3. Neural Networks

Two neural networks, the multilayer perceptron (MLP), and the radial basis function (RBF) were used to identify weeds and crops. The main characteristic of these models is their capacity for learning by example. This property means that, when using a neural network, there is no need to programme how the output is obtained given certain input; rather, examples are shown of the relationship between input and output, and the neural network will learn the existing relationship between them by means of a learning algorithm. Once the neural network has learnt to carry out the desired function, the input values can be entered and the neural network will calculate the output. 

The MLP neural model is a fully connected multilayer feed-forward supervised learning network trained by the back-propagation algorithm to minimise a quadratic error criterion. That property means that no values are fed back to earlier layers. The size of the MLP is described as size of input layer × size of hidden layer × size of output layer [34, 35]. In our case, the input layer is the annual set of spectral measurements taken from the spectroradiometer for cruciferous weeds, wheat, and broad beans. One hidden layer from 3 to 11 neurons was used for 2007, 2008, 2009, and 2010, and one output layer containing as many neurons as classes to which the samples are classified was used for every sampling year. 

The RBF is also a fully connected feed-forward neural network with an input layer, a hidden layer, and an output layer. The variables of the input and output layers were the same as for the MLP method. One hidden layer from 2 to 10 neurons was used for 2007, 2008, 2009, and 2010. The main differences between these two neural networks are that, in the RBF, the connections between the input and output layers are not weighted, and the transfer functions on the hidden layer nodes are radially symmetric [36]. The main difference between the STEPDISC and both neural networks is that the latter present a fitted function in an analytical form, where parameters are weights, biases, and network typology, whereas the STEPDISC produces a discriminant function (or a set of them) based on linear combinations of independent variables (i.e., spectral readings). 

The fitness of MLP and RBF for every classification model was determined by a hold-out cross-validation procedure, where the size of the training dataset was 3*n*/4 and *n*/4 for the test set, *n* being the full dataset in every sampling year. Consequently, the full dataset was randomly split into two datasets, and, after learning, the MLP or RBF model is run on the test set that provides an unbiased estimate of the generalisation error. SPSS application was used for STEPDISC, MLP, and RBF, and the classification performance of every method was evaluated. 

## 3. Results 

**Figure 1:**
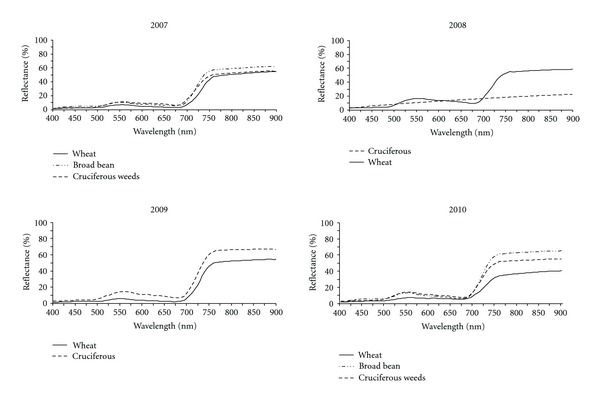
Mean hyperspectral curves for cruciferous weeds, and wheat and broad bean crops for every sampling year.

**Figure 2:**
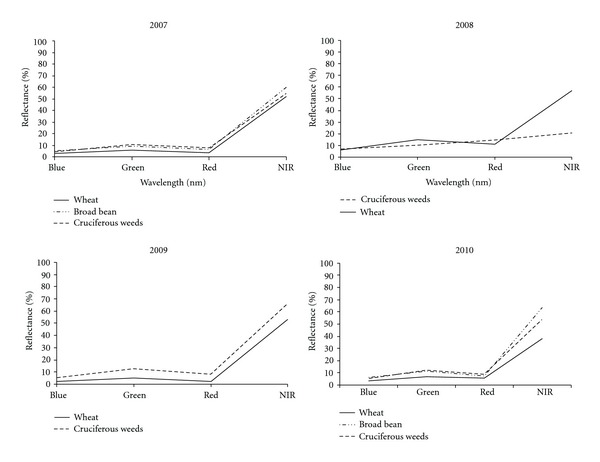
Mean multispectral curves for cruciferous weeds, and wheat and broad bean crops for every sampling year.

Mean hyperspectral and multispectral curves of cruciferous weeds, winter wheat, and broad bean crops obtained over four years are shown in Figures 1 and 2. Both graphs exhibited the characteristic peak in the green region of the spectrum at 550 nm (Figure 1) and green waveband (Figure 2) and the highest reflectance values in the near-infrared domain (from 760 to 900 nm) typical of green vegetation. There were apparent reflectance differences in all the wavelengths or multispectral bands for weeds and crops every year, suggesting potential for distinguishing weeds and crops on this basis. Figure 2 also shows that it might occasionally be helpful to use spectral vegetation indices to enhance these small spectral differences when they are not consistent. These results are in agreement with data obtained previously by other researchers [14, 16, 37, 38], who studied late-season weed discrimination when spectral differences between crop and weeds prevail, for example, when a flowering or still-green weed is present in a vegetative growth stage or early senescent crop. 

The classification results given in Table 2 were obtained from the STEPDISC analysis model for a different set of wavelengths (hyperspectral study) and wavebands or spectral vegetation indices (multispectral study) that were chosen on the basis of their order of entry into the STEPDISC procedure selection to discriminate between crops and weeds. The correct classification percentage for hyperspectral and multispectral analyses was 100% for 2008, being higher than 97.6% for the rest of years. A number of wavelengths ranging from 3 to 12 and along with the four multispectral bands and all of the spectral vegetation indices with a different order of entry were selected to develop every discriminant function for separating the spectra in the different years considered. 

The most frequently selected wavelengths were in the blue (405, 410, and 430 nm) and near-infrared (705 nm) parts of the spectrum. Wavelengths higher than 825 nm were not chosen for any of the discrimination models or years. While B/G, R/B, and RVI spectral indices were selected in every discriminant function for every year, B/G proved to be especially important: it was the first, second, or third variable entered into the discriminant functions in all years, indicating that it was crucial in classifying weeds and crops. The B band, the R/G ratio, and the ratios created from the combination of the NIR band with others (i.e., NIR/B and NIR/G spectral indices) also showed great potential for discrimination, as they were preferentially selected in 2007, 2009, and 2010. In each of the years studied, a small Wilks' lambda (near 0) was obtained, indicating the high discriminatory power of every set of selected wavelengths, wavebands, and vegetation indices or ratios. 

The correct classification percentage was 100% in 2007, 2008, and 2009 for MLP and higher than 98.1% for 2010, and, for RBF, was 100% in 2008 and higher than 80.4% for the rest years, when including input data from hyperspectral, and multispectral and spectral vegetation indices (Table 3). According to these findings, classification accuracy from hyperspectral signatures was similar to that from multispectral and spectral indices. Due to the fact that multispectral remotely sensed imagery from QuickBird satellite is cheap, covers a large amount of surface area, and is available worldwide, we present Table 4 to list the percentage of correct classifications for STEPDISC and MLP and RBF models for multispectral data from cruciferous weeds and winter crops for both the whole interval and selected indices as well as for every waveband and individual vegetation index. We found that MLP was the best method to distinguish crops and cruciferous weeds, with STEPDISC being second best; classification results from STEPDISC were more accurate than those from RBF for all years. MLP exhibited 100% classification accuracy for the NIR band and for most of the vegetation indices tested. All three models exhibited the best classification accuracy in 2008, followed by 2009 when only cruciferous weeds and winter wheat were included in the classification set. 

For clarity in the results, and because discriminant functions and MLP models were the most accurate, only a classification matrix using cross-validation for the STEPDISC analysis and the MLP model showing both classification accuracies lower than 100% is presented in Table 5. The values in the table provide the percentage of both correctly classified classes (accuracy) and misclassified classes (error percentage). Using the STEPDISC analysis, cruciferous weeds were always correctly classified in 2007, while 1% and 3% of cruciferous weeds spectral signatures were misclassified as wheat in 2009 and as wheat and broad bean in 2010. The lowest accuracy was for wheat in 2006, for which 3% and 5% of spectra were misclassified as broad bean and cruciferous weeds, respectively. Using the MLP model, in 2010, 2% and 1% of cruciferous weed spectra were misclassified as wheat and broad bean, respectively. 

## 4. Discussion 

Cruciferous weeds can considerably reduce the yield of wheat and legume crops. Because these weeds and crops are abundant in temperate regions of the world, more information about the distribution of weed patches is needed in order to carefully target herbicide use or other control strategies. The main objective of this research was to develop techniques to distinguish on-ground hyperspectral and multispectral field signatures of wheat, broad bean, and cruciferous weeds. The use of field data from different years and locations usually entails complications, due to exogenous factors. However, we aimed to analyse real field spectroradiometry data for further application of the results obtained in this study with the goal of using remotely sensed data to obtain cruciferous weed maps for site-specific control strategies at large scales (farming states or districts). Our results show that there are sufficient spectral differences between cruciferous weeds and crops to allow correct classification using both hyperspectral and multispectral measurements. The best overall classification was achieved with MLP for all years, followed by STEPDISC analysis and RBF. These findings are in close agreement with previous results from [35, 39] for weed and irrigated crops classification and when comparing two neural models and the discriminant statistical model. These authors showed that MLP achieved higher classification performance than STEPDISC analysis and RBF. The superior performance of MLP could be because the discriminant functions are based on linear combinations of the independent variables, whereas the neural network functions have network typology and their parameters include weights and biases. The RBF model is simple in typology and provided relatively high accuracy, although it required significantly more computations for a feed-forward output compared with the back-propagation MLP network. By contrast, the MLP function showed the highest classification accuracy with less computational requirements. Thus, MLP surpasses both STEPDISC and RBF in terms of accuracy, and it is also preferable to RBF in terms of computational demands. 

Our hyperspectral results suggest opportunities for the use of airborne hyperspectral sensors, such as CASI, which is user-programmable and capable of acquiring spectral data at up to 288 wavelengths, whereas our multispectral results indicate the possibility for use of multispectral satellites, such as QuickBird. STEPDISC analysis and the MLP neural network, when applied to spectral data collected from the ground, appear to be promising approaches for the classification of spectral signatures of cruciferous weeds, wheat, and broad bean crops. Future investigations will be essential to determine the potential of these techniques to distinguish and map this vegetation using CASI and QuickBird imagery taken when weeds and crops are at the specified phenological stages. 

CASI should be programmed with 30 or 12 wavelengths, rather than with the 288 available wavelengths in the cases of further use of STEPDISC or MLP models, respectively, in the visible and NIR spectral range. Our multispectral results show that the wavebands corresponding to QuickBird satellite imagery and to several spectral vegetation indices were capable of correctly classifying cruciferous weeds and crops. However, because classification accuracy from hyperspectral data (dozens of narrow wavelengths) was only slightly better than that from multispectral data (four wavebands and seven spectral vegetation indices) and because hyperspectral sensing is generally more expensive and covers a smaller surface area, we suggest that cruciferous weeds might be most efficiently distinguished using high-spatial-resolution QuickBird imagery. This approach could be an economical method for mapping broad-scale weed infestations to develop in-season postemergence site-specific management. This map-based approach would consider not only the appropriate spectral and spatial resolution for weed data acquisition but also the development of robust methods for analysis and delineation of management zones for further use. 

In addition, our results can also inform the potential use of image analysis for real-time site-specific weed management. Real-time monitoring and spraying consists of a weed control system that can simultaneously detect and control weeds on finer spatial scales using digital cameras or spectral or optical sensor systems (nonimaging sensors) from ground-based platforms. This strategy requires robust monitoring, processing techniques, decisionmaking, and spraying, while the vehicle is moving forward at a constant speed. Therefore, an algorithm classifying weeds and crops has to be powerful and flexible in a number of field situations to improve the decision making process. Some studies have applied fuzzy logic algorithms and artificial neural network classifiers to discriminate weed species in maize at the two- to five-leaf stage [40] and in sunflower at the four-leaf stage [41]. The success of their results is related to the performance of the image analysis process. 

Research to improve the potential of remote or proximal sensing for mapping weeds can greatly contribute to decisionmaking in SSWM, which is one of the essential goals of current European policy on the sustainable use of pesticides [42]. This policy includes such elements as reductions in pesticide applications and the utilisation of adequate pesticide doses, both of which are core tenets of SSWM. 

## 5. Conclusions 

Our results show that hyperspectral and multispectral signatures (spectral range of 400–900 nm) of cruciferous weeds, wheat, and broad beans taken under field conditions for 4 years can be classified with varying success using STEPDISC analysis and MLP and RBF networks. The MLP model was the most accurate, achieving 100% or nearly 100% correct classifications for all the years of our study. This model selected twelve wavelengths (480, 485, 490, 520, 565, 585, 590, 595, 690, 720, 725, 730 nm), three wavebands (B, G, NIR), and five spectral vegetation indices (B/G, R/B, R/G, NIR/B, RVI). The MLP neural network function could be considered for a future classification of hyperspectral or multispectral remotely sensed data for a map-based approach or for on-ground sensed data in the case of real-time-based site-specific weed management. However, differences in hyperspectral and multispectral classification results indicated that little advantage would be obtained using the more expensive airborne hyperspectral imagery. Therefore, multispectral high-spatial-resolution satellite imagery, such as QuickBird, or multispectral sensor systems from ground-based platforms (tractors, harvesters, robots) will be a useful next step to explore whether these kinds of imagery can potentially distinguish these weeds and crops. Successful spectral discrimination of weeds from crops would have several economical and environmental advantages: herbicide application on winter wheat could be reduced, thereby reducing costs, and tillage or hand-weeding in broad bean could be limited only to infested areas. This would improve the decision-making process for in-season postemergence herbicides (or other control strategies) according to current European agricultural and environmental policy.

## Figures and Tables

**Table 1:** Sampling years and spectral data acquisition information for cruciferous weeds and crops.

Years	Dates	Locations and number of fields	Crops
2007	April 11	Cañada*	Broad bean
		Galván*	Winter wheat
	April 14	Montalbán*	Winter wheat
2008	April 10	Lantejuela (two fields: A and B)	Winter wheat
2009	March 19	La Rambla (four fields: A, B, C and D)	Winter wheat
	March 20	Fernán Núñez (four fields: E, F, G and H)	Winter wheat
2010	April 21	Guadalcázar (two fields: A and B)	Winter wheat
		La Veguilla (two fields: A and B)	Broad bean
	April 22	Écija*	Winter wheat
		Castro*	Broad bean
		Espejo*	Broad bean

*Location with one field sampled.

**Table 2:** Hyperspectral and multispectral classification of cruciferous weeds, and wheat and broad bean crops using STEPDISC analysis according to the different sampling years.

Sampling years	Spectral input data	Wavelengths (nm), multispectral bands, and spectral vegetation indices selected	Wilks' Lambda	Exact-F	Overall classification (%)	Cross validation (%)
2007	Hyperspectral wavelengths	480, 405, 460, 705, 440, 680, 655, 595, 690, 515, 430, 445	0,000	244,5	99,3	98,0
	Multispectral bands and spectral vegetation indices	B, B/G, R/G, RVI, NIR, NIR/B, R/B	0,004	187,5	98,0	98,0
2008	Hyperspectral wavelengths	605, 690, 410	0,008	7387,5	100	100
	Multispectral bands and spectral vegetation indices	NIR/G, RVI, B/G, R/B	0,000	218990,5	100	100
2009	Hyperspectral wavelengths	725, 825, 815, 470, 430, 420, 410, 650, 665, 775, 540	0.131	517,8	99,2	99,2
	Multispectral bands and spectral vegetation indices	G, B/G, NIR/B, B, NIR/G, NDVI, R/B, R, RVI, R/G	0,111	681,9	99,3	99,1
2010	Hyperspectral wavelengths	735, 575, 485, 885, 410, 705, 525, 560, 750, 460, 405, 420	0,036	555,3	98,7	98,5
	Multispectral bands and spectral vegetation indices	B/G, R/G, NDVI, R/B, NIR/G, RVI, NIR/B, G, B, NIR, R	0,032	645,6	97,8	97,6

**Table 3:** Hyperspectral and multispectral classification for cruciferous weeds, and wheat and broad bean crops using MLP and RBF neural networks according to the different sampling years.

Neuronal networks	Sampling years	Input data	Importance of variables	Neurons of hidden layer	Neurons of output layer	Overall classification (%)
MLP	2007	Hyperspectral wavelengths (nm)	725, 720, 690	11	3	100,0
		Multispectral bands and spectral vegetation indices	B/G, R/B, B, R/G	4	3	100,0
	2008	Hyperspectral wavelengths	520, 585, 565	1	2	100,0
		Multispectral bands and spectral vegetation indices	NIR, G, NIR/B	6	2	100,0
	2009	Hyperspectral wavelengths	730, 595, 590	3	2	100,0
		Multispectral bands and spectral vegetation indices	B/G, G, NIR/B	5	2	99,4
	2010	Hyperspectral wavelengths	480, 490, 485	5	3	98,7
		Multispectral bands and spectral vegetation indices	B/G, R/G, RVI	8	3	98,1

RBF	2007	Hyperspectral wavelengths	705, 400, 710	10	3	88,7
		Multispectral bands and spectral vegetation indices	NIR/B, RVI, NIR/B	7	3	92,1
	2008	Hyperspectral wavelengths	620, 630, 605	4	2	100,0
		Multispectral bands and spectral vegetation indices	G	10	2	100,0
	2009	Hyperspectral wavelengths	610, 615, 655	9	2	94,8
		Multispectral bands and spectral vegetation indices	B/G, R/G, NIR/B	10	2	98,9
	2010	Hyperspectral wavelengths	415, 410, 420	10	3	80,4
		Multispectral bands and spectral vegetation indices	R/G, B/G, NIR/B	10	3	94,4

**Table 4:** Percentage of correct classification of cruciferous weeds, and wheat and broad bean crops for multispectral wavebands and spectral vegetation indices obtained from STEPDISC analysis, and from MLP and RBF neural networks.

		Wavebands (nm) and spectral vegetation indices											
Years	Classification methods	All bands (450–900) and spectral vegetation indices	B (450–520)	G (521–600)	R (630–690)	NIR (760–900)	NDVI	RVI	B/G	R/B	R/G	NIR/B	NIR/G
						(% correct classification)							
2007	STEPDISC	98.0	52.7	71.6	66.9	40.5	55.1	56.8	68.9	80.1	59.5	56.1	56.8
	MLP	100	55.9	70.4	65.5	60.0	72.0	74.2	55.9	82.1	71.4	75.0	56.7
	RBF	92.1	63.3	53.3	78.9	60.0	70.6	52.6	54.2	84.4	62.1	62.1	42.3

2008	STEPDISC	100	81.5	92.1	92.1	100	100	100	100	60.1	100	100	100
	MLP	100	79.5	94.6	88.2	100	100	100	100	81.8	100	100	100
	RBF	100	77.6	90.6	84.8	100	100	100	100	71.9	100	100	100

2009	STEPDISC	99.3	89.0	92.7	94.1	74.2	75.1	91.2	91.8	94.2	85.1	87.4	93.2
	MLP	99.4	90.5	94.3	95.6	80.1	87.1	94.9	90.3	94.5	86.0	93.3	95.9
	RBF	98.9	89.8	92.9	98.3	83.6	90.6	96.6	89.9	92.0	86.2	92.4	97.8

2010	STEPDISC	98.7	42.1	48.3	48.9	57.3	51.2	54.9	77.5	65.5	66.2	32.6	50.5
	MLP	98.1	58.1	60.1	55.9	63.2	61.6	63.4	75.8	79.1	67.8	54.9	66.2
	RBF	94.4	54.5	56.8	58.4	64.3	60.2	63.9	78.3	78.3	71.5	48.2	66.3

**Table 5:** Classification matrix obtained from STEPDISC analysis (from 2007 to 2010), and from MLP neural network (for 2010) considering cross-validation and using multispectral wavebands and spectral vegetation indices for cruciferous weeds, and wheat and broad bean crops.

Sampling years	Classification methods	Observed	Predicted spectra		
		spectra	Broad bean	Wheat	Cruciferous weeds
				(% of correct classification)	
2007	STEPDISC	Broad bean	100	0	0
		Wheat	3	92	5
		Cruciferous weeds	0	0	100

2008		Broad bean	—*	—	—
		Wheat	—	100	0
		Cruciferous weeds	—	0	100

2009		Broad bean	—	—	—
		Wheat	—	99	1
		Cruciferous weeds	—	1	99

2010		Broad bean	99	0	1
		Wheat	2	96	2
		Cruciferous weeds	2	1	97

2010	MLP	Broad bean	97	1	2
		Wheat	0	100	0
		Cruciferous weeds	1	2	97

*Data not sampled.
